# Strategic Co-Location in a Hybrid Process Involving Desalination and Pressure Retarded Osmosis (PRO)

**DOI:** 10.3390/membranes3030098

**Published:** 2013-07-04

**Authors:** Victor S.T. Sim, Qianhong She, Tzyy Haur Chong, Chuyang Y. Tang, Anthony G. Fane, William B. Krantz

**Affiliations:** 1 School of Civil and Environmental Engineering, Nanyang Technological University, 50 Nanyang Avenue # N1-1B-35, 639798, Singapore; E-Mails: stsim1@e.ntu.edu.sg (V.S.T.S.); qhshe@ntu.edu.sg (Q.S.); 2 Singapore Membrane Technology Center, Nanyang Environment and Water Research Institute, Nanyang Technological University, 639798, Singapore; E-Mails: thchong@ntu.edu.sg (T.H.C.); agfane@ntu.edu.sg (A.G.F.); 3 Department of Chemical and Biological Engineering, University of Colorado, Boulder, CO 80303, USA; E-Mail: krantz@colorado.edu

**Keywords:** desalination, water reuse, reverse osmosis (RO), pressure retarded osmosis (PRO), synergy, co-location

## Abstract

This paper focuses on a Hybrid Process that uses feed salinity dilution and osmotic power recovery from Pressure Retarded Osmosis (PRO) to achieve higher overall water recovery. This reduces the energy consumption and capital costs of conventional seawater desalination and water reuse processes. The Hybrid Process increases the amount of water recovered from the current 66.7% for conventional seawater desalination and water reuse processes to a potential 80% through the use of reclaimed water brine as an impaired water source. A reduction of up to 23% in energy consumption is projected via the Hybrid Process. The attractiveness is amplified by potential capital cost savings ranging from 8.7%–20% compared to conventional designs of seawater desalination plants. A decision matrix in the form of a customizable scorecard is introduced for evaluating a Hybrid Process based on the importance of land space, capital costs, energy consumption and membrane fouling. This study provides a new perspective, looking at processes not as individual systems but as a whole utilizing strategic co-location to unlock the synergies available in the water-energy nexus for more sustainable desalination.

## 1. Introduction

The technology discussed in this paper necessarily involves terminology that might not be familiar to some readers. Hence, a glossary of special terminology used in this paper is given in Appendix A.

Human population crossed a milestone of 7 billion people on 31 October 2011. With it came an opportunistic trend—urbanization through migration that is spurring the growth of cities in the 21st century. By 2015, more than 600 million people will live in approximately 60 megacities worldwide (those with 5 million or more inhabitants). Energy demand will escalate owing to the increasing development of industries and infrastructure. In fact, competition for natural resources such as food, water and energy are the next major issues that may lead to future cross-boundary conflicts [1]. To ensure a continuous supply of clean water, desalination and water reuse will increase [2]. In fact, there are many regions globally that practices both water reuse and seawater desalination concurrently such as Australia, Singapore and California, United States. By 2016, desalination capacity is forecast to increase to 89 million cubic meters per day while water reuse capacity is expected to grow to 79.5 million cubic meters per day [3]. Fortunately the current processes for desalination and water reuse still can be improved to reduce their energy consumption. Desalination has come a long way in reducing its energy consumption from nearly 16 kWh/m^3^ 40 years ago to the current 3–4 kWh/m^3^ for large desalination plants [4,5,6]. Energy consumption as low as 1.8 kWh/m^3^ has also been reported for a pilot scale system using new, high permeability seawater reverse osmosis (RO) membrane elements [6]. However, for the past decade significant improvements have been limited. Future improvements in power consumption will require radically new designs such as hybrid desalination processes or new high permeability dense membranes that mimic nature’s efficient way of desalination via aquaporin proteins [6,7]. 

**Figure 1:**
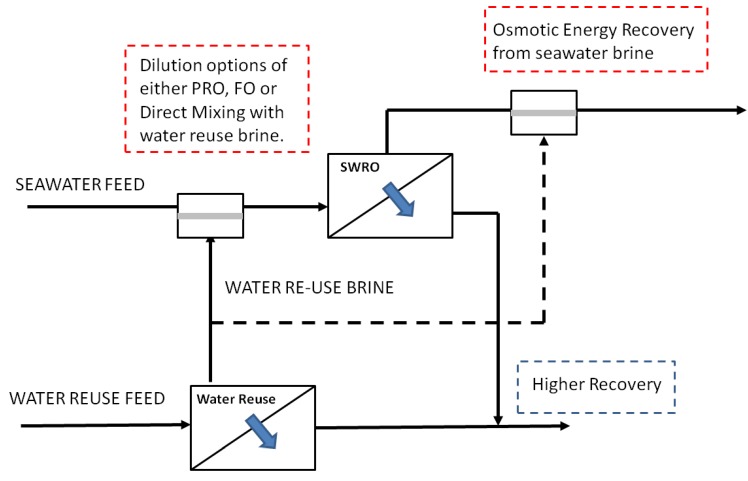
Proposed configuration of Hybrid Process. Adapted from Singapore-Netherlands Water Challenge 2011/2012 [8].

The current recovery for seawater desalination is within the range of 35%–55% [4]. This meant a large amount of pre-treated seawater feed and the associated chemicals is wasted together and the space footprint associated with the pre-treatment process not fully utilized. Even for water reuse plants where the objective is to conserve every drop of water, current membrane-based treatment is typically running at around 75%–80% recovery [4]. A proposed system for the production of industrial water referred to as the Hybrid Process, that uses Pressure Retarded Osmosis (PRO, see Appendix B for more details) coupled with seawater desalination and water reuse has been evaluated using the concept of strategic co-location [8] in an attempt to identify the possible synergies of the seawater desalination and water reuse processes. There are various designs for the proposed Hybrid Process. Focus has been placed on a design depicted in Figure 1 that concurrently dilutes the feed solution with impaired water for membrane-based seawater desalination to reduce the energy consumption for the desalination process and generates renewable energy via options of PRO, Forward Osmosis (FO) or direct mixing. This is followed by the use of the brine from the desalination process as the draw solution for a second PRO process to recover additional “osmotic” energy from otherwise perceived waste brine streams requiring proper disposal. Overall, a higher recovery is possible as compared to the seawater desalination and water reuse operating independently. The Hybrid Process helps to promote sustainable, economical brine disposal and *in situ* generation of renewable energy, which have been highlighted as some of the potential challenges facing the desalination industry [7]. However, this arrangement requires the strategic co-location of water reuse and desalination plants as part of urban city planning.

Prior study by other authors using a thermodynamic approach has found that the Hybrid process indeed has the potential to reduce the specific energy consumption of seawater desalination to well below the theoretical minimum work of separation [9]. In fact, the Hybrid Process of FO and RO integration has been gaining interest in recent years [5,10,11,12,13,14]. However, no attempt to calculate the potential savings quantitatively has been made that would be similar to a prior study done on a hybrid process of reverse electrodialysis (RED) and RO integration [15]. Therefore, the focus of this paper is to provide an engineering perspective for a conservative estimate of the cost/benefit for the various configurations of the Hybrid Process that has yet to be covered. An ancillary goal is to ensure minimal disruption of conventional processes and equipment while capitalizing on potential savings from synergistic coupling in terms of energy consumption and capital costs. The economics of desalination is attempted semi-qualitatively, as a scorecard for possible factors that affect decision making in the adoption of the Hybrid Process, and yet, customizable to fit different scenarios that would not be possible to contain in one single study.

This paper is organized as follows: Section 2 looks at the assumptions for the design considerations. Section 3 introduces the various possible configurations. The results of the cost analysis will be given and discussed together with a sensitivity analysis of the Hybrid Process and possible further improvements in Section 4. The conclusions are summarized in Section 5. 

## 2. Design Considerations

The hybrid design considers the need for strategic co-location of seawater desalination and water reuse plants. Additional piping will be required but could be minimal given the proximity of the hypothetical sites (assumed to be less than 2 km). The baseline comparison comprises of two plants: a conventional coastal seawater desalination using seawater RO membranes (SWRO) at 50% recovery (60 bar operating pressure) and a water reuse plant using brackish water RO at 75% recovery (10 bar operating pressure). In the following sub-sections, design considerations are given for the capacity and desalination plant configurations, forward osmosis (FO)/PRO operating conditions, membrane fouling and post-treatment improvements. These factors need to be considered in order to make cost projections for the hybrid design in comparison with the baseline. Table 1 summarizes the main design conditions with the justifications explained in Section 2.1, Section 2.2, Section 2.3.

### 2.1. Plant Capacity and Configurations

Water reuse is favored over seawater desalination as the primary means for water supply because it is the more energy efficient alternative [16]. The following conditions are assumed: the water reuse to seawater desalination capacity is 3:1 for the purpose of having sufficient water reuse brine for the Hybrid Process; for the purpose of calculation, values of 1 m^3^/s (86,400 m^3^/day) for SWRO and 3 m^3^/s (259,200 m^3^/day) for water reuse are chosen for the product water production rate; and, in order to estimate the osmotic pressure, sodium chloride concentrations of 0.04 M, 0.5 M and 1.0 M are used to simulate water reuse brine (assuming reclamation of secondary treated sewage effluent with a conductivity of 1100 μs/cm^2^ [17] at 75% recovery), seawater feed and seawater brine, respectively.

The brine from the water reuse process is used as the impaired water source for seawater dilution. It is employed either across membrane barriers such as FO and PRO or via direct mixing. Pre-treatment and brine disposal are assumed to have a total energy consumption of 1 kWh/m^3^ [4,5,6]. The energy required to overcome the osmotic pressure for SWRO is assumed to be 2 kWh/m^3^, with the use of isobaric energy recovery devices (ERD) [4,5,6]. Energy consumption for the brine disposal from SWRO is assumed to depend on the product of the brine concentration and the volume disposed. A reduction in the brine volume or concentration would lower the outfall height and reduce the energy required for proper brine disposal and mitigate the environmental impact. The energy consumption for water reuse is assumed to be 0.79 kWh/m^3^ based on 0.60 kWh/m^3^ for the water recovery system [18] and 0.19 kWh/m^3^ for pre-treatment of the feed [19]. A negligible contribution is assumed for the disposal of the water reuse brine that is discharged directly to the sea or estuary. 

Under the assumed conditions of plant capacities, the contributions to the overall energy consumption based on rate of product water are 75% and 25% from the water reuse (3 m^3^/s) and seawater desalination (1 m^3^/s) processes, respectively. Therefore, the specific energy consumption for the baseline of a conventional SWRO plant and a water reuse plant based on total product water using Equation (2) is 1.35 kWh/m^3^.

**Figure 2:**
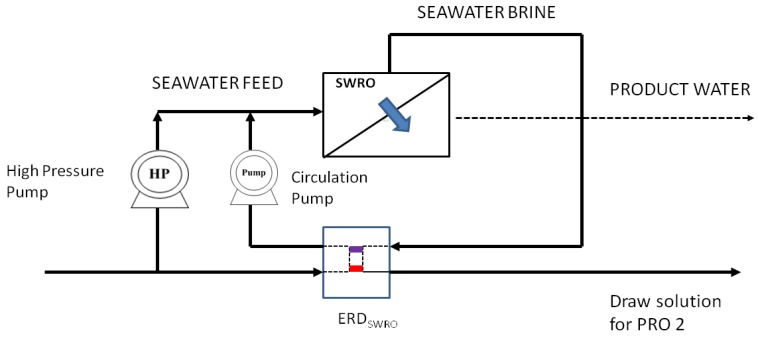
Seawater RO membranes (SWRO) process with isobaric energy recovery devices (ERD), ERD_SWRO_.

The capital cost of high pressure and circulation pumps, ERDs and the space footprint in the overall hybrid design are compared to the baseline of a conventional SWRO plant and a water reuse plant. Figure 2 shows total pre-treatment and high pressure pump capacities of 2 m^3^/s for SWRO and 4 m^3^/s for the water reuse plant. Any increase or decrease of capacity is expressed as a percentage of that specified for the baseline case. In addition, all capital costs are assumed to be amortized over a period of 25 years with the exception of the membrane elements to be discussed in Section 2.2. 

**Figure 3:**
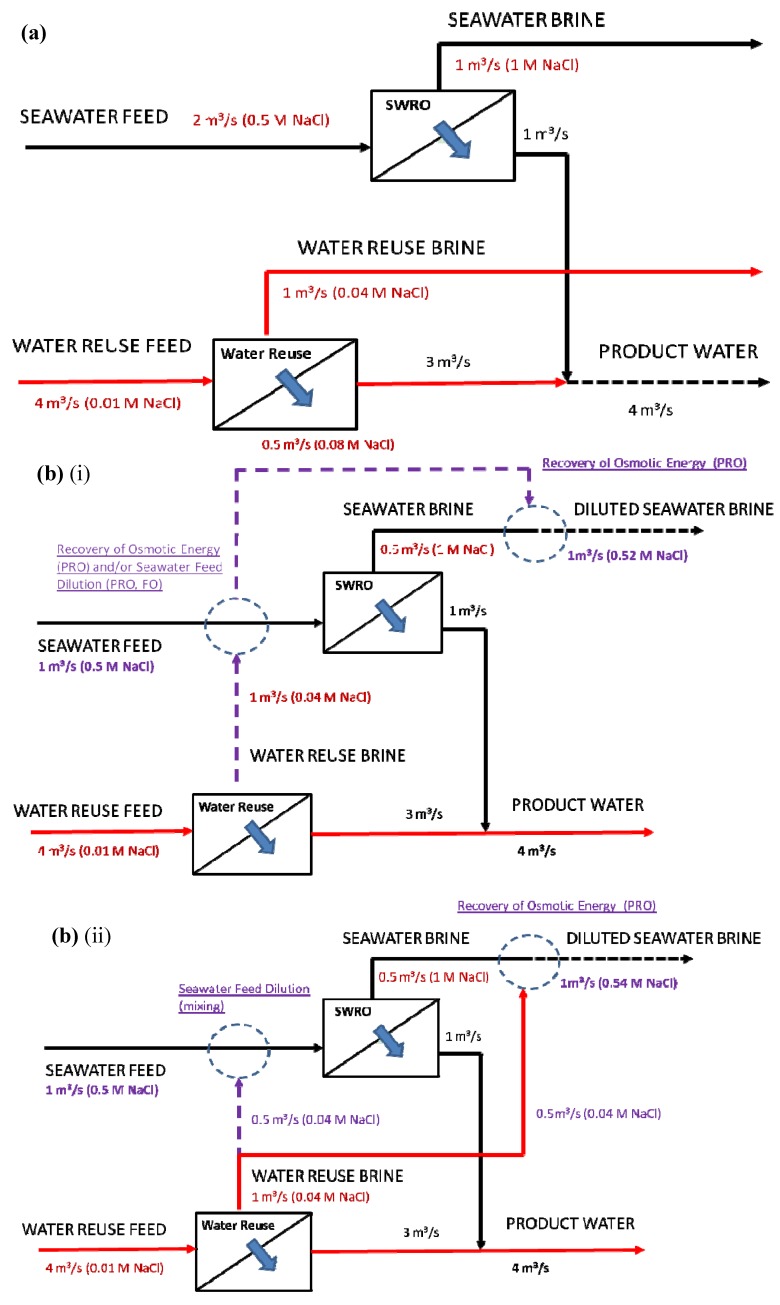
(**a**) Schematic of the baseline comprising a SWRO plant and a water reuse plant and; (**b**) Schematic of Hybrid Process Configuration(s): (i) Additional seawater feed dilution from impaired water sources using PRO or FO and (ii) direct mixing. All configurations achieve higher overall recovery from seawater desalination and water reuse since total feed source has reduced in terms of lesser amount of seawater feed required. Major differences between Figure 3, Figure 4, which are the schematics of the baseline comparison and the different configurations of the Hybrid Process, respectively, are highlighted in purple color.

There are two configurations for the Hybrid Process: configuration (i) that uses PRO or FO and configuration (ii) that uses direct mixing. Figure 3 shows schematics of the baseline and the various possible configurations of the Hybrid Process with the key differences between the baseline and the different configurations highlighted in purple. The key differences will be discussed in Section 4. 

### 2.2. FO/PRO Operating Conditions and Considerations

The recovery from the FO and PRO process is fixed at 50%. The overall water recovery from water reuse is assumed to be 87.5% or an additional 0.5 m^3^/s of product water allowed by the diluted seawater feed. The recovery of osmotic energy for all configurations is achieved with PRO technology. Although the theoretically maximum power density is available at half the osmotic pressure [20], the operating pressures for PRO processes are deliberately chosen at 40% the osmotic pressure of the draw solutions. By using seawater and seawater brine as the draw solutions, the fluxes for PRO are assumed to be 18 LMH and 15.84 LMH [21], respectively. This results in a power density of 5 W/m^2^ at an operating pressure of 10 bar and 8.8 W/m^2^ at an operating pressure of 20 bar by using seawater and seawater brine as the draw solutions, respectively, for the PRO processes. For the FO process, only seawater is used for the draw solution for which a flux of 25 LMH is assumed [21,22,23]. For the estimation of the membrane module footprint, conventional 8-inch spiral wound RO modules are used as the reference. Since dual membrane spacers are needed for the feed and draw solution sides employed in either FO or PRO, the 8-inch FO or PRO spiral wound module is assumed to provide only 70% of the 400 ft^2^ (*i.e.*, 25.9 m^2^) of the area available for SWRO spiral wound elements that require only a single spacer (for the brine channel). It is also assumed that the PRO and FO elements are priced competitively with conventional 8-inch spiral wound RO elements. The capital costs for all membrane elements are amortized over five years.

**Table 1:** Hybrid Process Design Conditions.

Design Conditions									Values	Units
Coastal Seawater Desalination Plant										
	Feed Flow Rate								172,800	m^3^/day
	Recovery [4]								50%	–
	Product Flow Rate								86,400	m^3^/day
	Energy for Water Treatment [4,5,6]								2	kWh/m^3^
	Energy for Pretreatment & Brine Disposal [4,5,6]								1	kWh/m^3^
	Specific Energy Consumption								3	kWh/m^3^
Water Reuse Plant										
		Feed Flow Rate							345,600	m^3^/day
		Recovery [4]							75%	–
		Product Flow Rate							259,200	m^3^/day
		Energy for Water Treatment [18]							0.60	kWh/m^3^
		Energy for Pretreatment & Brine Disposal [19]							0.19	kWh/m^3^
		Specific Energy Consumption							0.79	kWh/m^3^
Pressure Retarded Osmosis (PRO)										
(a) Draw: Seawater; Feed: Water Reuse Brine										
		Flux [21]							18	LMH
		Power Density							5	W/m^2^
		Nominal Membrane Surface Area of 8 Inch Spiral Wound Modules							18.13	m^2^
(b) Draw: Seawater Brine; Feed: Concentrated Water Reuse Brine										
		Flux [21]							15.84	LMH
		Power Density							8.8	W/m^2^
		Nominal Membrane Surface Area of 8 Inch Spiral Wound Modules							18.13	m^2^
Forward Osmosis (FO)										
(a) Draw: Seawater; Feed: Water Reuse Brine										
		Flux [21,22,23]							25	LMH
		Nominal Membrane Surface Area of 8 Inch Spiral Wound Modules							18.13	m^2^
Energy Recovery Devices (ERDs) [24,25]										
		Isobaric Efficiency							95%	–
		Non-isobaric Efficiency							70%	–

**Figure 4:**
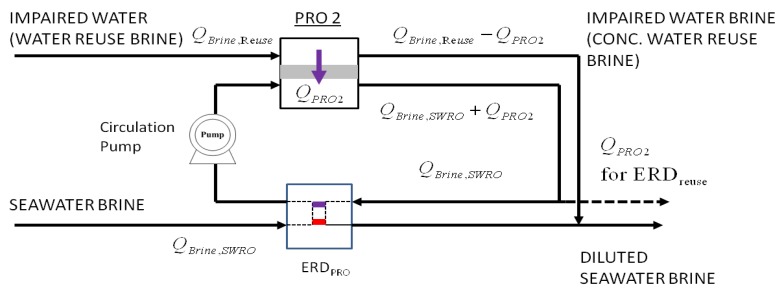
PRO process of high efficiency, adapted from [26].

When using seawater as the draw solution in the PRO mode (PRO 1), it is more efficient to use the osmotic power generated directly to pressure a portion of the feed, which should be close to 100% efficiency, rather than recovering the osmotic power generated and then re-pressuring the feed for SWRO. Before using seawater brine as the draw solution in the PRO mode (PRO 2), it is more efficient to recover the energy from the pressurized brine, which is after the SWRO process, at an operating pressure of 60 bar to re-pressurize the feed solution for the SWRO process using isobaric ERD, which is termed as ERD_SWRO_. Figure 2 shows the SWRO process incorporating the isobaric ERD, ERD_SWRO_. This can be compared to the alternative method of de-pressuring the brine using a non-isobaric ERD before re-pressuring the draw solution at an operating pressure of 20 bar again for PRO (PRO 2). This results in an efficiency loss of 25% or more if the alternative method is employed since isobaric ERD is capable of 95% efficiency, whereas non-isobaric ERD has, at most, 70% efficiency [24,25]. To improve the overall efficiency of PRO, the additional use of isobaric ERD, which is termed as ERD_PRO_, in pressurizing the draw solution for PRO 2 does eliminate the need for high pressure pumps [26]. Figure 4 shows the PRO process capable of high efficiency. The use of PRO processes may be counter-intuitive for capital cost savings but the reduction in seawater salinity decreases the amount of seawater feed required for the SWRO process. Therefore, there are savings from the reduction of capacity for the ERDs and high pressure pumps. This is better illustrated in the schematics for the different configurations shown in Section 3. 

**Figure 5:**
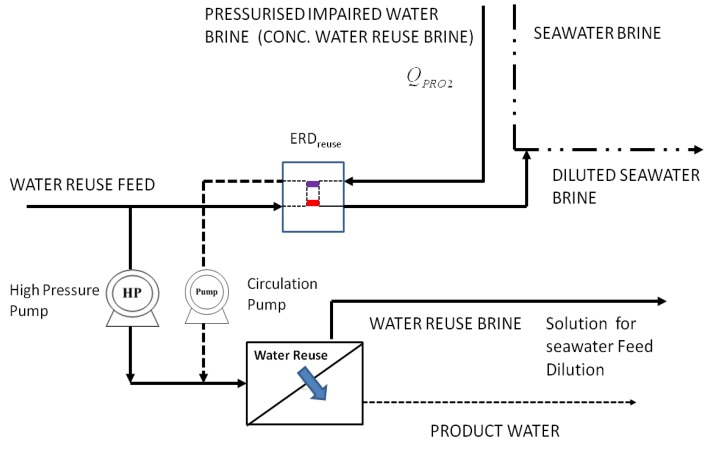
Water reuse process with non-isobaric ERD, ERD_reuse_.

The osmotic energy recovered from PRO 2 can be used in various forms. For example, turning turbines to generate electricity is the simplest form but is also the least energy efficient. Using the osmotic energy in isobaric ERD is the most energy efficient but is suitable only if the SWRO is carried out in a staged operating variation that allows the operating pressure of a particular stage to be the same as the outlet pressure of the draw solution in the PRO process. In this paper staged operation in SWRO is not considered. Therefore, the osmotic energy recovered from PRO 2 is used to reduce the energy consumption of the water reuse process at 70% efficiency using a non-isobaric ERD, which is termed as ERD_reuse_. Figure 5 shows how the non-isobaric ERD, ERD_reuse_, is being employed in the water reuse process.

### 2.3. Membrane Fouling and Post Treatment Improvements

Membrane fouling is likely to occur in all membrane processes but is assumed to be controllable using conventional mitigation strategies. For water recoveries above 87.5% for a water reuse process, membrane scaling can be expected but is controllable if back-washing is possible for the PRO modules [27]. Novel PRO flat sheet membrane development has the potential to allow back-washing due to a robust membrane support [28]. Use of either FO or PRO as a primary membrane barrier to dilute the seawater feed with water reuse brine prior to the SWRO process, which is essentially a secondary membrane barrier, should be adequate to match the quality of potable water from conventional water reuse processes [17]. When a primary membrane barrier is not used for the dilution of the seawater feed prior to the SWRO process, ultraviolet (UV) post-treatment of the desalinated water could be employed to improve the quality. However, it is noted in a pilot scale demonstration plant in Japan using water reuse brine for seawater dilution prior to the SWRO process in the absence of a primary membrane barrier [29], there was no information on whether deterioration in the water quality occurred. Therefore, the UV treatment system might not be necessary. It is included to highlight possible improvements; however, the energy consumption for the UV system is not considered here. 

### 2.4. Decision Matrix

Without the possible consideration of improving recovery and solely focusing on the amount of product water produced, *Q_Product_*, the following considerations associated with incomplete recovery, which are not possible to express in monetary terms such as (1) the water cycle, *Q_Feed_* being a limited resource; (2) any possible environmental degradation caused by the additional usage of chemicals and brine disposal; and (3) space footprint are not being recognized. A key idea is not to just look at desalination on the monetary sense but possibly taking all likely factors into consideration, which is introduced in this work as a customizable scorecard to aid in decision making. However, any realistic future development is likely governed by economics and any improvements in the specific energy consumption would have to be considered together with the whole costs of development based on both conventional factors such as capital costs and operational expenses and also unconventional factors such as space footprint and carbon tax.

A decision matrix in the form of a customizable scorecard is introduced to aid in choosing a hybrid system appropriate for a particular scenario. Expressing the cost in monetary terms might appear to be the most straightforward metric to quantify the relative merits of the various configurations. Indeed, for seawater desalination, an average costing approach can easily be made based on the data from many plants globally. Unfortunately, there are insufficient data to assess the cost variations in the Hybrid Process to account for differences such as the availability of land in a space-constrained area. For example, prior studies do not differentiate between the needs of a seawater desalination facility for municipal use and an off-shore oil rig for which space is extremely valuable. Clearly an alternative metric for cost is needed to assess hybrid processes for water desalination and treatment. A decision matrix in the form of a scorecard to take into account different priorities is proposed here. The following parameters are considered in the scorecard: energy consumption, capital cost, space footprint and fouling tendency of the entire process. Each section is given points in the range of 1 to 4 where 1 is the most desired outcome and 4 the least desired outcome among the available choices (*i.e.*, three variations of the Hybrid Process and the baseline comparison). Each section could be given a certain importance weighting factor to compute the overall scorecard. The lowest score would be the most suitable configuration for the chosen scenario. 

The assignment of the weighting factors would vary depending on the country but is not done here owing to the inability of assigning these for any meaningful global scenario. For example, in Australia land would be weighted much lower than membrane fouling and overall energy consumption. Energy consumption in Australia would be weighted even more heavily if a carbon tax were put in place of if environmental factors such as sustainability must be considered [30,31]. The space footprint could be given the highest weighting factor for the scorecard if the Hybrid Process is being considered for land-scarce countries. Since most countries are not endowed with fossil-fuel sources, the relative merits of the Hybrid Process are strongly affected by high energy prices. Therefore, energy consumption could be assigned a relatively high weighting factor as well. Membrane fouling in the SWRO process, which affects the long term energy consumption, could also be assigned a similar weighting factor as energy consumption. The remaining factor that includes the total capital cost of pumps, ERDs, membrane elements, seawater intakes and pre-treatment, and brine disposal could be allocated the remaining weightage. The Hybrid Process configuration could then be selected based on using such weightings in the scorecard to aid decision making. Obviously, the weightings could be adjusted for other scenarios and the scorecard is an adjustable tool to take into account possible factors that affects decision making to adopt the Hybrid Process since some factors are not able to be expressed in monetary terms.

## 3. Hybrid Process Configurations

The configurations of the Hybrid Process are analyzed with respect to their energy consumption, expected capital cost and membrane space footprint. There are three variations depending on whether PRO, FO or direct mixing is used to achieve the required dilution factor for the seawater feed. The total osmotic energy generated is normalized with respect to 1 m^3^ of product water from the SWRO. The amount of osmotic energy generated is calculated using the product of the total amount of permeate from the impaired water source and the operating pressure of the PRO module minus the loss from the recovery of the mechanical energy used to pressurized the inlet feed of the PRO device at 95% efficiency as depicted by Equation (1) and illustrated by Figure 4. For example, for the FO 1 variation, the amount of osmotic energy available for ERD_reuse_ is 0.13 kWh/m^3^ calculated from the product of 0.25 m^3^/s and 20 bar with a 5% efficiency loss by ERD_PRO._ A detailed comparison of the various configurations is discussed in Section 4.



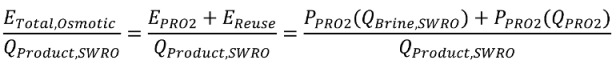
(1)
Where *E_Total,Osmotic_* is the total osmotic energy available from the PRO 2 process.

  *E_PRO2 _*is the amount of energy required from *E_Total,Osmotic_* to run the PRO 2 process continuously.

  *E_Reuse_* is the amount of energy available from *E_Total,Osmotic_* to be recovered by the non-isobaric ERD, ERD_reuse_.

  *P_PRO2 _*is the operating pressure for the PRO 2 process, which is at 25 bars.

### 3.1. Various Configurations

There are three variations, namely FO, PRO or direct mixing, of the water reuse brine with seawater feed to achieve a dilution of 50%. This is an important advantage since the water reuse brine is now used as a feed source. The total feed source is now reduced from 6 m^3^/s to 5 m^3^/s. Therefore, the total recovery has increased to 80% as compared to 66.7% for the baseline. 

**Figure 6:**
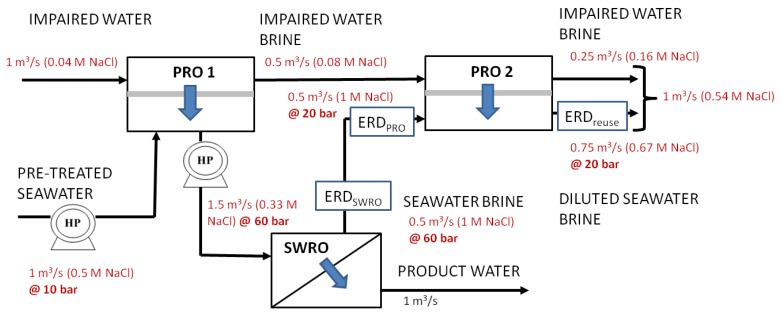
Configuration pressure retarded osmosis (PRO) 1, diluted seawater water feed using impaired water (water reuse brine) with dual-stage recovery of osmotic energy.

**Figure 7:**
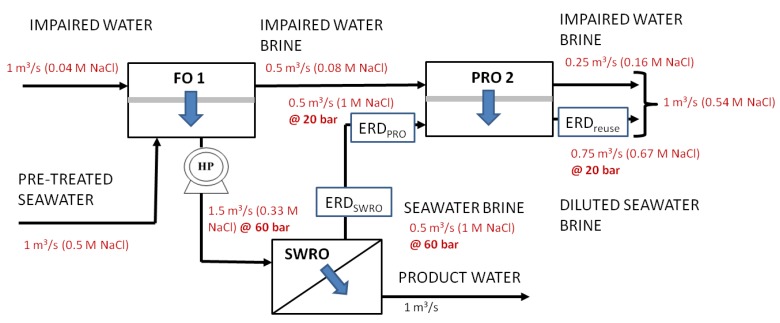
Configuration forward osmosis (FO) 1, diluted seawater water feed using impaired water (water reuse brine) with an FO process and single-stage recovery of osmotic energy.

**Figure 8:**
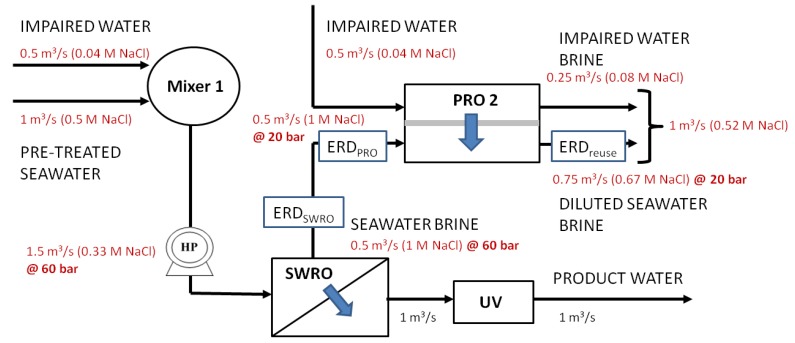
Configuration Mixer 1, diluted seawater water feed using impaired water (water reuse brine) with direct mixing and single-stage recovery of osmotic energy.

Figure 6, Figure 7, Figure 8 show the variations (PRO 1, FO 1and Mixer 1) incorporating the key concepts of higher recovery from seawater desalination, osmotic power recovery from seawater brine and seawater feed dilution using PRO, FO or a direct mixing process. The total amount of osmotic energy available from PRO 1 variation is 0.23 kWh/m^3^, whereas only 0.13 kWh/m^3^ is available for variations FO 1 and Mixer 1. If the osmotic energy generated in the PRO 2 process is used to reduce the energy consumption for producing water reuse, the amount of osmotic energy recovered from all variations is 0.09 kWh/m^3^. This results in a reduction of energy consumption to 0.76 kWh/m^3^ for the production of water reuse for all variations. The capacity reduction of 25% for the seawater feed source, 50% for seawater feed pre-treatment and a nearly 50% reduction in the seawater brine concentration result in reducing the energy consumptions to 2 kWh/m^3^ for SWRO for variations FO 1 and Mixer 1. There is an enhanced energy reduction to 1.86 kWh/m^3^ for the SWRO in using variation PRO 1 incorporating the osmotic power recovered from PRO 1. 

In summary, the total energy consumption is 1.04 kWh/m^3^ for the PRO 1 variation and 1.07 kWh/m^3^ for variations FO 1 and Mixer 1 based on the total product water. The membrane footprint or inventory, increased by 2780, 3860 and 2195 8-inch spiral wound elements for FO 1, PRO 1 and PRO 2, respectively. There is a reduction of 50% in the capacity required for the pre-treatment of seawater intake and a reduction of brine disposal for all variations, as the amount of pre-treated seawater feed has reduced 50% to 1 m^3^/s and the total concentration of brine to be disposed has also reduced by nearly 50% to 0.52–0.54 M or close to the salinity of seawater feed. There is no additional capacity requirement for the isobaric ERD, ERD_SWRO_ for the SWRO process as the reduction of the ERD_SWRO _has now been utilized by the isobaric ERD, ERD_PRO_ for the PRO 2 process, but an increase of 0.25 m^3^/s for non-isobaric ERD, ERD_reuse_ in the water reuse process is required for all variations. The reduction in the seawater feed required results in a 25% decrease in the capacity requirement for the high pressure pumps for SWRO with the exception of a 25% increase in the PRO 1 variation caused by the pressurization of the draw solution for PRO 1, which is before the SWRO. Note that the least increase in the number of spiral wound elements occurs for variation Mixer 1. However, there may be serious membrane fouling implications for this arrangement. Dilution of the seawater feed using either FO or PRO as a primary membrane barrier would result in less fouling when compared to the conventional SWRO process under similar operating conditions. Direct mixing on the other hand may give mixed results. Direct mixing dilutes the seawater feed and water reuse brine assuming that the composition of the foulants is different. Clearly different mixtures of foulants and ionic backgrounds need to be investigated with respect to the fouling tendency of the SWRO. In view of the lack of information on this, it is assumed that the fouling behaviour of the SWRO is the worst due to the incompatibility of the water chemistry that may cause both organic fouling and scaling problems. 

### 3.2. Summary of Energy Consumption

**Figure 9:**
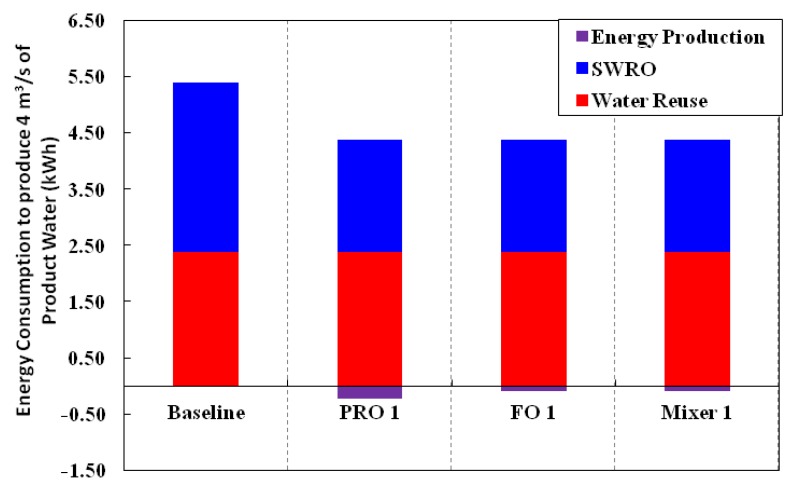
Comparison of the total energy consumption for the baseline and various configurations of the Hybrid Process.

**Figure 10:**
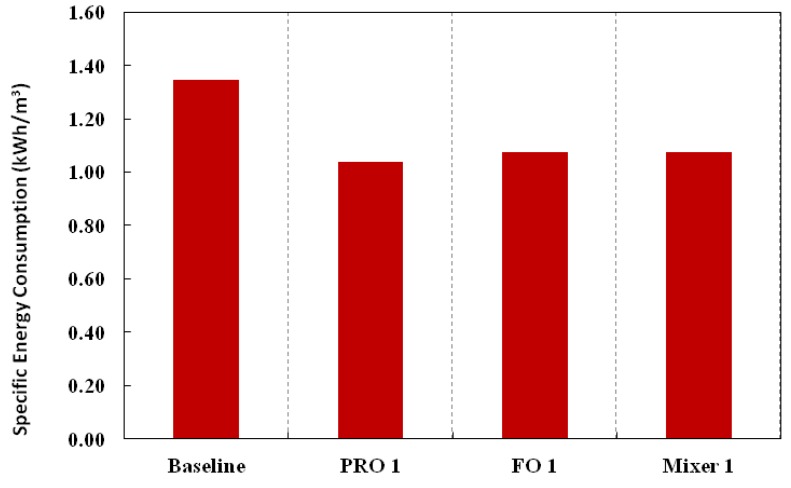
Specific Energy Consumption of the various configurations expressed in the conventional way in terms of unit volume of product water.

Figure 9, Figure 10 summarize the total energy consumption, the main contributors to the reduction in energy consumption for the SWRO process and the specific energy consumption, respectively, for the baseline (conventional SWRO and water reuse) and different variations. For detailed breakdown of the calculations, please refer to Table A3 in Appendix D. Figure 9 clearly shows the main decrease in energy consumption is due to the reduction of energy consumption in the SWRO process. Specific energy consumption decreases for all variations of the Hybrid Process as compared to the baseline. This is not surprising as the total energy consumption decreased for the same amount of product water produced with a higher overall recovery from 66.7% to 80%.

## 4. Results and Discussion

### 4.1. Summary of Comparison

**Figure 11:**
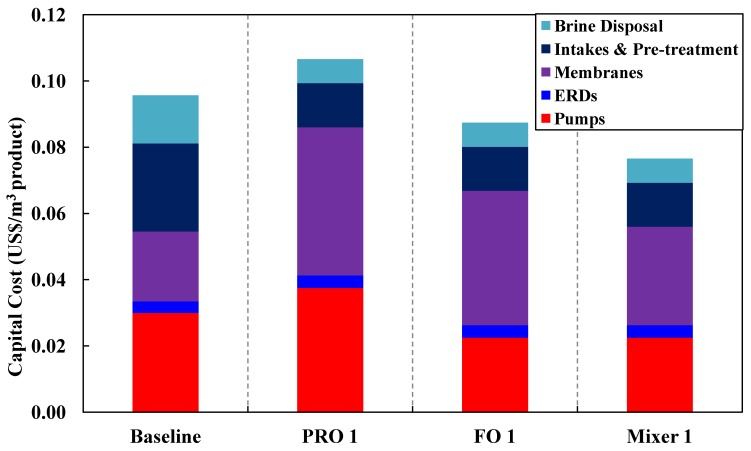
A summary of the capital costs for the various configurations.

**Figure 12:**
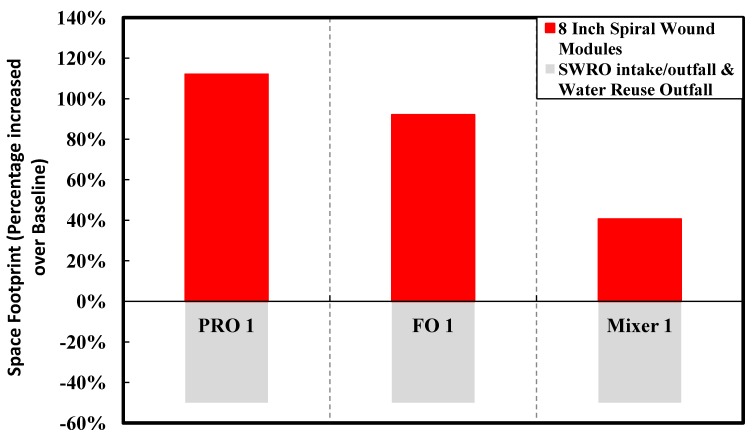
A summary of the space footprint considerations of the various configurations expressed as a percentage of the baseline.

A quantitative comparison of the different configurations for the capital costs and space footprint is shown in Table A2 in Appendix C.3. The breakdown of the capital cost determination is given in Appendices C.1 and C.2. Figure 11, Figure 12 summarize the capital cost and the space footprint, respectively, for the baseline and various configurations of the Hybrid Process. Table 2 show the decision matrix based on the findings of Figure 11, Figure 12. Any configurations of the Hybrid Process will result in lower energy consumption for which the reduction can range from 20% to 23% based on total product water as calculated in Table A3 in Appendix D and illustrated in Figure 10. The Mixer 1 variation, which mixes seawater directly with water reuse brine, is of particular interest. Although the space footprint for the additional spiral wound modules increased by 41%, savings on multiple fronts such as seawater intakes, pre-treatment, pumps and energy consumption were achieved. This makes Mixer 1 one of the most attractive designs for implementation in land-scarce countries such as Singapore. However, membrane fouling can be a major concern in this design. The FO 1 variation is another attractive hybrid design for which membrane fouling is of less concern. Unfortunately, for PRO 1 variation the increase in capital cost and space footprint appear to outweigh the benefits of reduced energy consumption. Both the FO 1 and Mixer 1 variations are sufficiently attractive to be explored in more detail for comparison to the baseline of conventional seawater desalination and water reuse process. Moreover, the configurations allow the discharge of waste streams near the salinity of seawater as indicated in Figure 6, Figure 7, Figure 8. This assessment provides consideration motivation for further research in scaling up the Hybrid Process. In order to validate the assumptions used in this study, research is needed on the membrane fouling, fouling control strategies, quality of desalinated water for the different Hybrid Process configurations relative to established water reuse standards. Moreover, the possibility of an operating prototype should be considered. 

**Table 2:** Decision Matrix Scorecard.

Weighing Factors	Baseline	Configurations		
	SWRO/Newater	B–PRO 1	B–FO 1	B–Mixer 1
**Overall Energy Consumption ^3^ (kWh/m^3^)**	4	1	2	2
**Membrane Fouling Tendency ^1^**	3	1 ^a^	1 ^a^	4 ^a^
Total Capital Cost ** (US$/m^3^)	3	4	2	1
**Space Footprint ^2^**	1	4	3	2

** Excludes capital cost of post-treatment and other costs (construction/engineering); ^1^ Considering only with reference to conventional SWRO; ^2^ Spiral wound modules take up much more space and more expensive as MF/UF pre-treatment modules per m^2^; Therefore, membrane savings in terms of cost and space for feed source pre-treatment is considered not significant; ^3^ There is negligible difference in the energy consumption between FO 1 and Mixer 1; ^a^ Seawater feed dilution with a membrane barrier would result in lower fouling tendency than the conventional SWRO baseline due to the dilution effect but with direct mixing, membrane fouling would be expected to be the worst due to incompatibility of water chemistry that may cause mixture of organic fouling and scaling problems.

### 4.2. Sensitivity Analysis of Hybrid Process

**Figure 13:**
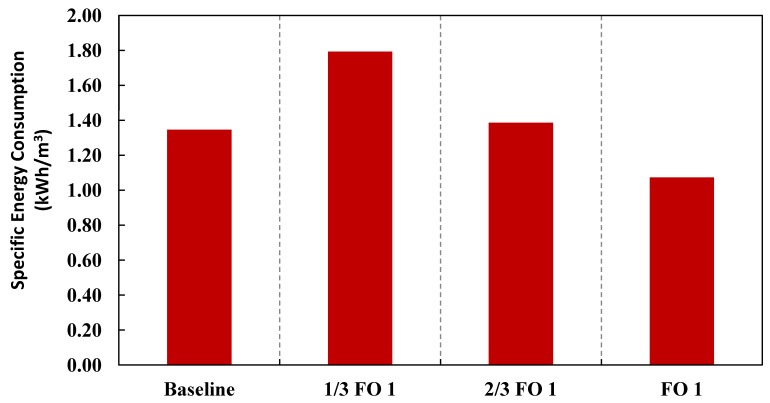
Specific Energy Consumption of the various capacities of the water reuse plant expressed in the conventional way in terms of unit volume of product water if water reuse plants were reduced by 1/3 and 2/3, labeled as 1/3 FO 1 and 2/3 FO 1, respectively.

**Figure 14:**
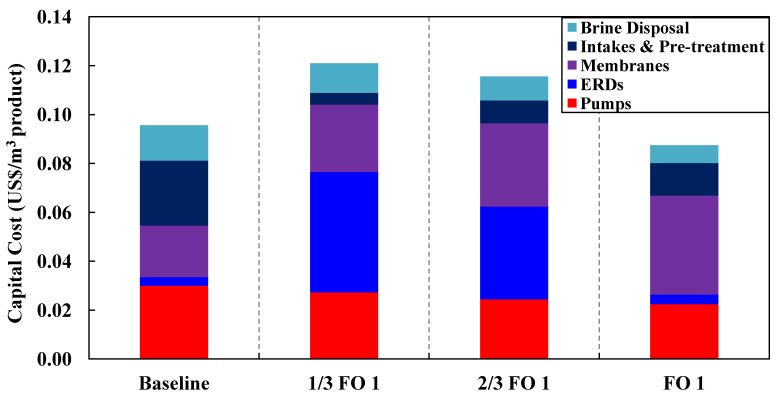
Overall capital costs of the various capacities of the water reuse plant if water reuse plants were reduced by 1/3 and 2/3, labeled as 1/3 FO 1 and 2/3 FO 1, respectively.

This study serves as a conservative and simplified method for evaluating the various configurations of the Hybrid Design to consider the synergistic effects of seawater feed dilution, osmotic power recovery and higher overall recovery. Conservative in the sense that a hypothetical scenario based on average process values is assumed as outlined in the design considerations that can be potentially affected by other considerations such as the type of feed source for both the water reuse and SWRO plants. The feed source could affect the operating pressure, fouling and scaling behavior, pre-treatment and brine disposal methods. Moreover, a critical aspect of the Hybrid Process is the quantity of impaired water available for the dilution of the seawater feed and the possible reduction of the seawater feed pre-treatment and brine disposal that makes the Hybrid Process a successful alternative. This could be an issue since water-stressed countries/areas usually rely on SWRO to make up for the shortfall in the water supply. As such, there is the distinct possibility that water-stressed countries might not have sufficient impaired water sources. In any event, the main concern is the available capacity for the water reuse plant to be three times that of the SWRO plant. A sensitivity analysis was carried out on the configuration FO 1 of the Hybrid Process and assumed that the capacity of the water reuse plant is reduced by one to two folds, labeled as 1/3 FO 1 and 2/3 FO 2, respectively. The specific energy consumption and capital costs are re-analyzed as Figure 13, Figure 14. Without sufficient impaired water sources, the possible reductions of the seawater feed pre-treatment and brine disposal as well as the dilution of the seawater feed are limited. As might be anticipated in the absence of a sufficient amount of impaired water in configuration 1/3 FO 1, both the specific energy consumption and capital cost would increase significantly as shown in Figure 13.

### 4.3. Further Improvements

Separately, there are potential increased savings in energy consumption, which have not been explored in this initial study since it would have resulted in a more complex evaluation. For example, an increase in the feed temperature from 30 to 35 °C would reduce the osmotic pressure and thereby reduce the energy consumption by approximately 10%, although there would be a trade-off of a 0.5% decrease in the salt rejection owing to the increased membrane permeability [32]. By running the SWRO process in stages, the first stage of which might resemble a brackish water RO process, it is possible to reduce the operating pressure in the SWRO by 33%. A similar study on an integrated design FO/RO process with conventional two-pass RO suggests that a lower operating pressure for the RO process would result in a 23% improvement in the energy efficiency [5]. Moreover, an isobaric ERD could be used if the choice of operating pressure for the first stage is similar to the pressure of the draw solution in the outlet of the PRO 2 (e.g., 20 bar in this study). This would increase the efficiency of recovering the osmotic energy generated from the current 70% to as much as 95%. Indeed, there could be potential to decrease the energy consumption by 50% or more for producing desalinated water via a more thorough consideration of the potential synergistic processes in the Hybrid Design using a co-location strategy for a seawater desalination plant and a water reuse plant. 

## 5. Conclusions

This paper has evaluated various configurations of the Hybrid Process to assess the synergistic effects of seawater feed dilution, osmotic power recovery and a higher overall recovery in comparison with conventional designs of seawater desalination and water-reuse plants. The introduction of a decision matrix in the form of an adjustable scorecard that can be adapted to accommodate different priorities is proposed to complement conventional costing norms for the evaluation of the Hybrid Process. A conservative assessment indicates that the Hybrid Process offers a potential energy reduction of 20%–23%. This is coupled with a potential total capital cost reduction of 8.7%–20% for the two optimal configurations. However, this requires an increase in the number of spiral wound reverse osmosis elements that increases the footprint by at least 41% relative to a conventional seawater desalination plant. This could be mitigated somewhat by the decrease in footprint associated with a reduction in the size of the pumps, seawater intakes, and equipment for brine disposal and pre-treatment. Exploring these additional potential savings is clearly an area for further study.

## Appendix A (Glossary of Terms)

- Water reuse plants: Water reclamation plants utilizing brackish water reverse osmosis membranes.
- SWRO plants: Seawater desalination plants utilizing seawater reverse osmosis membranes.
- Product water: Water that permeates in either a SWRO plant or a water reuse plant.
- Water reuse feed: the feed source used for a water reuse plant.
- Water reuse brine: the retentate for a water reuse plant.
- Seawater feed: the feed source used for a SWRO plant.
- Seawater brine: the retentate for a water reuse plant.
- Specific Energy Consumption: Amount of energy required to produce unit volume of product water.
- Impaired water: Water reuse brine when used in the Hybrid Process configuration to dilute the feed source of SWRO plants or seawater feed.
- Impaired water brine: The retentate from the hybrid process involving Forward Osmosis (FO) and Pressure Retarded Osmosis (PRO) utilizing impaired water.
- Industrial water: non-potable water used for industrial purposes.
- Outfall: a pipeline or tunnel that discharges seawater or water reuse brine to the sea.
- Outfall height: the height a brine plume must reach to ensure proper dispersal of brine.

## Appendix B (PRO State-of-the-Art)

Forward Osmosis (FO) occurs when fresh water and saline water are placed on opposite sides of a semi-permeable membrane that allows water to be drawn naturally from the freshwater side to the saline side by the osmotic pressure difference across the membrane. In Pressure Retarded Osmosis (PRO), which is essentially an FO process, the saline side is moderately pressurized. The difference between PRO and FO is that chemical potential energy from the difference in osmotic pressure can be recovered mechanically using the pressurized water to do useful work such as powering electrical generators [33]. Loeb and co-workers pioneered the PRO technology [34,35,36,37] that has generated renewed interest in recent years [20,21,29,33,34,35,36,37]. In early PRO studies, the use of conventional RO membranes having thick support layers resulted in very low power densities [35,38]. However, recent developments in osmotic membranes and processes have made PRO considerably more attractive [21,33,39,40,41,42,43,44,45,46,47,48,49].

A minimum power density of 5 W/m^2^ is required to make PRO commercially viable [50] since a large amount of membrane area is needed at low water fluxes and low power densities. Until recently the best reported PRO membranes had a power density of only 5.64 W/m^2^ [36], thereby barely meeting the target 5 W/m^2^. A recent invention has achieved a power density of 15.2 W/m^2^ and able to withstand high operating pressure of 15 bars using a thin-film membrane incorporating nanofibres [51]. This new membrane offers the potential to move PRO from the laboratory to a viable commercial technology.

## Appendix C (Capital Cost Simulation)

### C.1. Capital Cost Calculations

Capital cost estimation is performed for a 25-year lifetime SWRO at a capacity of 100,000 m^3^/day. Typically there are five main sections in an SWRO plant, namely seawater intake, pre-treatment, RO system, post-treatment, and brine disposal [52,53,54]. Therefore, the total capital cost, CC_T_, can be written as:

(5)

where *CC_OC_*, *CC_SI_*, *CC_Pre_*, *CC_RO_*, *CC_Post_*, *CC_BD_*, denote the costs of others including construction and engineering costs, seawater intake, pre-treatment, RO system, post-treatment, and brine disposal, respectively.

In general, two types of seawater intake systems are available, the open intake and the beach-well method. The open intake system has the advantage of low investment cost but requires more pre-treatment of the raw water before it is fed to an RO system (which requires a Silt Density Index—SDI of <3.5); whereas the beach well system has the advantage of less polluted seawater but higher investment cost [55]. Since the intake system and pre-treatment system are inter-dependent, a simple correlation to estimate the combined capital cost of the intake and pre-treatment system is used here [52,56]:

(6)

where *Q_Feed_* (m^3^/h) is the total feed flow rate.

The capital cost of the RO system, CC_RO_, consists of the high pressure pump, energy recovery device, RO element, and pressure vessel:

(7)

where *CC_HPP_*, *CC_ERD_*, *CC_E_*, and *CC_PV_*, denote the costs of high pressure pump, energy recovery device, RO element, and pressure vessel, respectively.

The cost for the high pressure pump is a function of the feed flow rate, *Q_F_*, and pressure, *P_F_* [bar] and is approximated by [56]:

(8)

where *N_HPP,A_* , *N_HPP,B_* , and *N_HPP,C_* are the number of high pressure pumps in category A, B, and C, respectively. Category A, B, and C, refer to feed flow rates of 450 m^3^/h, 200–450 m^3^/h, and less than 200 m^3^/h, respectively. The cost for an energy recovery device, e.g., an exchanger device used to generate power from the pressure gradient between feed inflow and concentrate outflow, is estimated as follows [57]:

(9)

where *Q_brine_* (m^3^/h) is the total flow rate of brine. The total cost for the membrane element, *CC_E_*, is estimated as [58]:

(10)

where *N_E _*is the total number of RO elements.

This includes the fixed cost for membrane replacement that is assumed to have a lifetime of five years. The total cost for pressure vessel, *CC_PV_*, including 10% of cost of the plumbing and fittings is estimated as [59]:

(11)

where *N_PV_* is the total number of pressure vessels into each of which seven membrane elements can be fitted.

Post-treatment of the RO product water includes degasification, pH adjustment, and disinfection by chlorination and addition of corrosion inhibitor. It is estimated to be *CC_post_* = $4,000,000 for a SWRO plant capacity of 100,000 m^3^/day [59].

The cost of brine disposal depends on the brine characteristics, level of treatment before disposal, disposal method, environment, and brine volume [60]. Several methods are available for brine disposal, which include direct sea discharge, deep well injection and evaporation ponds. It is estimated that *CC_BD_* = $10,000,000 for a 100,000 m^3^/day plant, which includes the construction of injection wells and discharge pipelines of 4500 ft [59].

The other costs, *CC_OC_*, include the engineering and construction cost, which is assumed to be 10% of total direct cost, and the indirect cost, which is assumed to be 27% of the total direct costs.

### C.2. RO Data Calculations

In order to perform the cost analysis it is essential to have information on the RO data such as flow and pressure of feed (*Q_Feed_*, *P_Feed_*) and concentrate (*Q_Brine_*, *P_Brine_*) for sizing of the pre-treatment apparatus, high pressure pumps and lines, pressure exchanger, brine disposal pump and the number of membrane elements. The performance of the RO system is estimated using the commercially available software, IMdesign, by Hydranautics, a company of Nitto Denko. The required input parameters include plant capacity (*Q_P_* = 100,000 m^3^/day), recovery, seawater properties (assumed to be 0.50 M NaCl equivalent), membrane type (SWC4+ from Nitto Denko is selected and properties are summarized in Table A1), number of elements per vessel (assumed to be 7), number of passes (single pass is used in the simulation since the use of a high rejection membrane, overall rejection of >99% can be achieved), membrane life span (assumed to be 5 years), and fouling factor (assumed to be 0.80, which gives an annual flux decline of 4.4%). The total number of elements is fixed at *N_E_*, 6300 elements (or the number of pressure vessels, *N_PV_*, is 900). Based on the total feed flow *Q_F_*, the number of pumps required can be calculated:

*N_HPP,A_**= Q_Feed_*/450
(12)

*N_HPP,B_**=* (*Q_Feed_* − 450*N_HPP,A_*)/200 if 200 < (*Q_Feed_* − 450*N_HPP,A_*) < 450
(13)

*N_HPP,C_**=* (*Q_Feed_* − 450*N_HPP,A_*)/200 if (*Q_Feed_* − 450*N_HPP,A_*) < 200
(14)

**Table A1:** Properties of seawater reverse osmosis membrane (Nitto Denko).

Membrane Element	SWC4+
Membrane Active Area	**400 ft^2^ (37.1 m^2^)**
Membrane Polymer	**Composite Polyamide**
Configuration	**8–inch Spiral Wound**
Permeate Flow	**6500 gpd (24.6 m^3^/day)**
Salt Rejection	**99.8%**
Max. Applied Pressure	**1200 psig (8.27 MPa)**
Max. Feed Flow	**75 GPM (17.0 m^3^/h)**
Minimum Concentrate/Permeate Flow	**5:1**
Max. Pressure Drop per Element	**10 psi**
Maximum Feed Water SDI (15 mins)	**5.0**

### C.3. Breakdown of Capital Cost Calculations

**Table A2:** Quantitative Comparison of total capital costs and space footprint for the different configurations and the baseline of a conventional SWRO plant.

Capital Cost and Space Footprint	**Baseline**	**Configurations**					
	SWRO	PRO 1 ^3^	(% change)	FO 1 ^4^	(% change)	Mixer 1 ^5^	(% change)
Capital Cost of Pumps (US$/m^3^)							
High Pressure Pumps	0.030	0.037	(25%)	0.022	(−25%)	0.022	(−25%)
Capital Cost of ERDs ^6^ (US$/m^3^)							
Isobaric	0.0035	0.0035		0.0035		0.0035	
Non-isobaric (Water Reuse ERD only for PRO 2)	–	0.0003		0.0003		0.0003	
	0.0035	0.0038	(8.6%)	0.0038	(8.6%)	0.0038	(8.6%)
Capital Cost of Membranes ^7^ (US$/m^3^)							
	0.021	0.045	(112%)	0.041	(92%)	0.030	(41%)
Capital Cost of Intakes and Pre-treatment (US$/m^3^)							
	0.027	0.013	(−50%)	0.013	(−50%)	0.013	(−50%)
Capital Cost of Brine Disposal (US$/m^3^)							
	0.015	0.0073	(−50%)	0.0073	(−50%)	0.0073	(−50%)
**Total Capital Cost ** (US$/m^3^)**							
	0.096	0.1066	(11%)	0.0874	(−8.7%)	0.0765	(−20%)
**Space Footprint**							
8′′ spiral wound elements ^1^	5,405	11,460	(112%)	10,380	(92%)	7,600	(41%)
SWRO intake/outfall and Water Reuse Outfall ^2^	4 m^3^/s	2 m^3^/s	(−50%)	2 m^3^/s	(−50%)	2 m^3^/s	(−50%)

* For overall energy consumption and circulation pumps, the baseline comparison includes water reuse process; ** Excludes capital cost of post-treatment and other costs (construction/engineering); ^1^ 8′′ spiral wound RO modules of 37 m^2^ each for 1 m^3^/s capacity at flux of 18 LMH is 5405 elements.; ^2^ SWRO intake/outfall and water re-use outfall space footprint is not considered in the matrix scorecard since it is usually placed reasonably far out at sea; ^3^ 6055 8′′ spiral wound PRO elements; ^4^ 2780 and 2195 8′′ spiral wound elements for FO and PRO, respectively; ^5^ 2195 8′′ spiral wound PRO elements; ^6^ Capital cost of ERDs is based on the simultation done using equations found in Appendix B. Isobaric ERDs are around 20% more expensive than non-isobaric ERDs [61]; ^7^ 8′′ spiral wound PRO and FO elements are assumed to be priced competitively with RO elements. Cost of Pressure vessels are not included as they generally account for less than 10% of total cost of membrane modules over the plant’s 25 years useful lifetime.

## Appendix D (Energy Consumption)

**Table A3:** Total energy consumption for baseline (Water Reuse and SWRO) and different variations.

Breakdown of Specific Energy Consumption	Baseline		Configuration-PRO 1		Configuration-FO 1				Configuration-Mixer 1	
	Water Re-use	SWRO	Water Re-use	SWRO	Water Re-use		SWRO		Water Re-use	SWRO
**Volume of Product Water (m^3^/s)**	3	1	3	1.0	3		1.0		3	1.0
**Volume of Total Feed Water ^1^ (m^3^/s)**	4	2	4	1.0	4		1.0		4	1.0
Pressurized Feed Water (m^3^/s)	4	2	4	1.5	4		1.5		4	1.5
**Volume of Brine (m^3^/s)**	1	1	1		1				1	
Concentration of Brine (M NaCl)	0.04	1	0.54		0.54				0.52	
**Energy Consumption (kWh/m^3^)**										
Entire system for water recovery	0.60	2	0.60	1.50	0.60		1.50		0.60	1.50
Pre-treatment/Brine Disposal	0.19	1	0.19	0.50 ^a^	0.19		0.50 ^a^		0.194	0.50 ^a^
**Energy Recovery from PRO ^2^ (kWh/m^3^)**	N.A.		0.23		0.09				0.09	
**Specific Energy Consumption (kWh/m^3^)**										
In terms of Product Water	1.35		1.04		1.07				1.07	

^1^ Total feed water and pressurized feed water may not be equal as there is additional SWRO feed from impaired water source (Water reuse brine); ^2^ Normalized with respect to product water from SWRO but used to offset energy consumption of water reuse; ^a^ There is a 50% reduction in total brine concentration and amount of pre-treated seawater feed.

## Nomenclature

*CC_T_*: Total capital cost ($)
*CC_SI_*: Capital cost of seawater intake ($)
*CC_Pre_*: Capital cost of pre-treatment ($)
*CC_RO_*: Capital cost of RO system ($)
*CC_Post_*: Capital cost of post-treatment ($)
*CC_BD_*: Capital cost of brine disposal ($)
*CC_OC_*: Capital cost of others including construction and engineering ($)
*CC_HPP_*: Capital cost of high pressure pump ($)
*CC_ERD_*: Capital cost of energy recovery device ($)
*CC_E_*: Capital cost of RO element ($)
*CC_PV_*: Capital cost of pressure vessel ($)
*Q_P_*: Plant capacity (m^3^/h)
*Q_Feed_*: Total feed flow rate (m^3^/h)
*Q_Product_*: Total product water flow rate (m^3^/h)
*Q_Brine_*: Total flow rate of brine (m^3^/h)
*E_Total,Osmotic_*: Total energy consumption (kWh)
*E_Spec_*: Specific energy consumption (kWh/m^3^)
*E_PRO2_*: Energy required to run the second PRO process (kWh)
*E_Reuse_*: Energy recovered from the second PRO process for the water reuse process (kWh)
*P_Feed_*: Feed pressure (bar)
*P_Brine_*: Brine pressure (bar)
*N_HPP,A_*: Number of high pressure pumps in category A (dimensionless)
*N_HPP,B_*: Number of high pressure pumps in category B (dimensionless)
*N_HPP,C_*: Number of high pressure pumps in category C (dimensionless)
*N_E_*: Total number of RO elements (dimensionless)
*N_PV_*: Total number of pressure vessels into each of which seven membrane elements can be fitted (dimensionless)
